# Paranoia as It Relates to Homicide1Read at the Fourth Pan-American Medical Congress, held at Panama, January 2-6, 1905.

**Published:** 1905-02

**Authors:** James Wright Putnam

**Affiliations:** Professor of Nervous Diseases, University of Buffalo


### BUFFALO MEDICAL JOURNAL.

VOL. XLIV.—LX. FEBRUARY, 1905. No. 7

### ORIGINAL COMMUNICATIONS.

### Paranoia as It Relates to Homicide.[1]

BY JAMES WRIGHT PUTNAM, M. D.,
Professor of Nervous Diseases, University of Buffalo.

THERE is no form of insanity which is of greater importance in criminal jurisprudence than paranoia. This statement is made because the paranoiac remains so often undiagnosticated until some act of violence, which results in the serious injury or death of his victim, calls the attention of medical men to his condition.

The examination of such criminals requires not only a painstaking study of the prisoner's history as it relates to his victim, but must include inquiry likewise into his heredity, his youth and, indeed, his whole life up to the time of the examination. The diagnosis of other types of insanity is comparatively easy. The recognition of paranoia is frequently extremely difficult; and oftentimes the demonstration of it to the satisfaction of public, judge, and jury is impossible even in well marked cases.

Lest I should be accused of exaggeration, I may refer to the case of Prendergast, the assassin of Carter H. Harrison, Mayor of Chicago, as recorded by H. M. Bannister. Prendergast was born in Ireland, and came to this country as a baby; his father was an inebriate, and several of his parental ancestors were insane. His mother was a strong woman, with no bad heredity that was ascertained. In early childhood he sustained a head injury by falling that made him unconscious for several hours. This was followed by more or less headache. As a boy he showed peculiarities, was very irritable, and did not care for the companionship of other boys. He went to school for a few

1. Read at the Fourth Pan-American Medical Congress, held at Panama, January 2-6, 1905.

years, and made very good progress. He became a newsboy and did quite as well as such boys generally do, but was rarely on good terms with the other boys. As he grew older he delivered daily papers on a somewhat secluded route, and did this work to the satisfaction of all. About the age of 15 he began to develop delusions of persecution; thought the other newsboys were combined against him, that they were making misrepresentations about him, and that his mother and brother were also against him and constantly trying to do him harm.

A little later, there was much agitation in the Chicago papers about the dangers of railroad grade-crossings and the necessity of stopping the loss of life by track elevation. Then Prendergast became possessed of the delusion that he was God's appointed agent to bring about this important work. To do it, he conceived the idea that he must be made the corporation counsel of Chicago. As soon as Mayor Harrison was elected he applied to him. After Hon. A. Krauss had been appointed, he called upon him and several times demanded the office as his, by right of being the Almighty's agent. At the time of the assassination he went in the early evening to the Mayor's residence and made the same demand. Being refused, he shot Mr. Harrison, and immediately thereafter went to the police station and gave himself up. At the station he was regarded as insane, and at first it was determined to send him to the Detention Hospital for the Insane. At the police station Prendergast insisted that he did it; that he was the divinely appointed agent to elevate the railroad tracks, and in order to do it properly he must be corporation counsel. The mayor refused to comply with his request, and the Lord had commanded him to remove him. He seemed sorry enough, but said he must do as the Almighty demanded. In various interviews he always admitted the killing, and always justified it by his delusions; he seemed to be very sorry that it had to be done. Upon other topics he talked as well as could be expected with his limited education, showing a good memory and emotional control. He had numerous stigmata of degeneracy. After two jury trials he was condemned and in due time hanged. No postmortem was permitted.

A definition of paranoia is essential, as it has been frequently misapplied. Paranoia, literally translated meaning "close to understanding," the term first applied by Mendel, is synonymous with the German primare verructheit, the French delire chronique a evolution systematique, and with the English terms monomania and chronic primary delusional insanity.

It is almost unanimously conceded that paranoia is nearly always due to inherited structural weakness of the nervous system. In fact, so strongly does Berkley of Johns Hopkins believe this that he says, "For my own part I have never seen a paranoiac in whose case a full and complete history could be obtained, that did not have a hereditary history of drunkenness, of family neuroses, or actual insanity."

With this strong position some able observers disagree, notably Mendel and Magnan. They maintain that only paranoia of early development should be considered as one of the hereditary degenerative type; and that those who have reached middle life before the disease develops should be regarded as non-hereditary. The Italian school of psychiatrists generally maintain that paranoia may be primary in some subjects, and secondary in others; that when it is primary in an individual it succeeds a generalised insanity in his ancestors; when it is secondary the insanity is confined to one individual. (Regis.)

American psychiatrists generally are committed to the view that paranoia is a primary psychosis founded on a hereditary basis. It may first manifest itself in middle life under the influence of various causes. These may be slight and of short duration; serious mental shocks, or simply the long continued wear and tear of life; the battle against poverty and want; the stress of society; the complications of business, domestic infelicity, and the like.

The development of paranoia in one cursed with neuropathic ancestry is gradual from earliest childhood. The future paranoiac is either above or below the average in the early accomplishments of talking, walking, or the use of hands. As the child passes into youth, physical peculiarities, which mark them from their fellows, often become apparent. Mentally, it is noted that they are either unusually seclusive, irritable, dreamy, or introspective. Although they are often studious, they seldom are exact, so that their progress in science and mathematics is less satisfactory than in art, language, and history.

When the duties and responsibilities of manhood and womanhood are assumed, their inability to resist the strain of life's discipline is manifested in various ways. If plans fail, and endeavor does not meet with success, then the suspicions of the individual against others are aroused. Self is never blamed. The patient becomes more and more under this domination, until from suspicion he passes into systematised delusions of persecution and keeps aloof from his friends, from whom he conceals his delusions.

Hallucinations of hearing usually accompany the disease. The history of the paranoiac often includes the story of wandering from place to place to avoid conspirators, and vain attempts to escape from the persecuting voices. Other patients are not secretive. They make confidants, and tell of their persecutions and explain their strange behavior. Owing to the continuation of delusions and hallucinations for many years, about one-third of the patients pass into a stage of grandeur. This stage is marked by delusions of changed personality. Because he is chosen for some great mission, he is persecuted and the conspirators are attempting to thwart him. In this stage environment, education and natural ability have much to do with the development of various types. For example, the politician believes in his call to power, and the fact that he does not attain it is laid at the door of the wicked, unpatriotic, or unappreciative. The disappointed inventor is possessed with the delusion that the patent office is in league against him. The religious enthusiast passes into a state of religious exaltation, and believes himself called upon to redeem mankind from the fetters of sin.

During all this time the paranoiac may retain his reasoning powers to a marked degree. He may engage in his business and attend to the daily routine of life, and conversation on other topics is often well and logically sustained. Analytic power with literary ability is not uncommon, this latter being well marked in Peterson's case, the author of "The Piling of Tophet." This book is an autobiography written by a paranoiac who, previous to his admission to the hospital, had attempted suicide with a revolver, and who also had delusions that the people of the village were acting upon him by magnetism, spoke disparagingly of him, and were conspirators against his peace. Of this autobiography, extracts of which appeared in the *American Journal of Psychology,* 1889, Peterson said: "I believe no better idea of the typical form of paranoia can be obtained than by its reading."

It is a graphic picture of the steady evolution of the malady, a remarkable self-dissection of the soul's anatomy. I must deny myself the pleasure of quoting more than the preface of this manuscript. It is sufficient to illustrate the literary ability of this paranoiac at a time when he was fully under the domination of his delusions.

This work is given to the public as a lunatic's defense of his position. Every effort I have made hitherto to come to an understanding with my fellow-men, on things which I see to proceed from them, and which give my life its whole shape, has drawn out nothing more than blank denials of all knowledge of the things I spoke of. Now, it is impossible for me to reduce my thoughts to the bounds which others have been willing to concede. The object of this little autobiography is to show the form and consistency of the thought that is in my mind.

I present my evidence to the tribunals of last resort, the public and the press, and ask them to try the case and render their verdict. Have I a right to my thought, or have I not? If not, where am I deceived? If I have, why is not mine the true thought of all men?

From my own cases in medico-legal practice I have selected two paranoiacs indicted for murder in the first degree, to illustrate the phenomena of this disease.

No. 1. A boy aged 19, of good family, without previous quarrel suddenly and without warning shot and killed a man in broad daylight, in a busy street. He was immediately seized, disarmed, and taken to jail. The boy talked freely of the shooting, and maintained that he was justified in doing so; that this man had frequently said to people that the boy was a bastard; that when he went away to a distant state and tried to obtain work at his trade it was refused him, and he knew at once the man had sent on word that the bastard was looking for a job. He went to a hotel in this strange city where two of the waitresses laughed, and he knew at once that they did so because this man who was persecuting him had told them he was a bastard. He went to sleep in his room and he could hear different voices as they passed, saying, "The bastard is in there," and could hear them speak and call his mother, the mother of the bastard.

Of course, there was no arguing with the boy that his victim did not know these people and could not possibly have informed them. He was confident he had punished justly the defamer of his mother, and that he had done right. He was angry at the thought that his lawyer should call him insane, and wished to be defended on the ground that he had done right, and that any right-minded and right-thinking man would have done as he did.

After three days of interviewing him and observations and taking testimony of his friends and neighbors, the experts for the people, Dr. Ford, of Utica, Dr. MacDonald, of New York, and the writer, reported to the District Attorney that the prisoner was insane and that the trial should not go on. A commission was appointed, the prisoner was declared insane and was sent to Mattewan, the institution for insane criminals of New York, where he has been for seven years.

The previous history of this boy was that of the average school boy. He was as good a pupil as most of his class. After leaving school he went into his father's store, learned the trade of watchmaker, and spent a large part of his time at work repairing watches. Gradually it was noticed that he withdrew from social gatherings, that he would not talk with people, and that he spent much of his time by himself. In the last year before the shooting he made two trips away from home, saying that he did not wish to work there longer. He returned both times, saying they were against him.

In deciding that he was a paranoiac we came to the conclusion that he knew the general difference between right and wrong, and that he thoroughly understood the consequence of the act of murder; but that this particular murder he believed to be right. When he was examined by the commission and declared insane by them, he expressed himself as much disappointed; that as he had done right in killing this man he should be tried and justified. He had no fear of conviction. I have heard of him from time to time since he has been at Mattewan. His delusions are the same. The statements are often repeated, that he did right, that he was glad he did it, and would always say so.

The other case of homicide by a paranoiac was studied by me as the chairman of a lunacy commission appointed by the court. The facts were these:

No. 2. The prisoner in July last had a slight argument with his employer, and suddenly, and without warning, stabbed him to death. He also stabbed a woman who tried to interfere. After the murder he disappeared and was not heard of for five months. He then returned, and meeting one of his friends, said he had come to get a job. He was arrested and committed to jail to await trial. The fact that he returned, and his peculiar behavior in the jail, resulted in the appointment of a lunacy commission. The prisoner was of good build and appearance. He presented none of the stigmata of degeneracy. The history as given by him, and by persons who had known him was as follows: he received an ordinary common school education, but never associated much with people, because he thought they laughed at him, and did not like him. About five years ago when working for a farmer, a telephone was put into the house and his suspicions were aroused. He had little to do with his fellows, always preferring to be by himself. Suddenly he left his employer, without giving a reason other than the telephone messages. He went to Atlantic City, and from city to city along the coast down to Florida, working a short time in each place, then suddenly leaving, without any other reason than the one that the telephones bothered him.

After continually changing his place of residence, he finally returned to his native town seeking employment. In this town he was hired by a farmer, and did his work satisfactorily. He frequently complained to the housekeeper, that he did not like to have people talking about him and telephoning about him. One day after a dispute about the time of milking, he announced his intentions to leave at once, and asked for his wages. In the discussion he attacked his employer, and killed him.

To me he explained his going away as having nothing to do with the crime; that he had planned to go before; that he had often been to Atlantic City; that he went to get work, and to get away from the people who always telephoned about him. People always seemed to know who he was and what his business was. He came back after being away five months, because he did not know the man was dead. He did not know that he was wanted. If he had been wanted, they knew by telephone just where he was all the time, and could get him at any time. So he came back and was going to work, when he was arrested.

This man knew murder was a crime, and was punishable by death. He was able to reason correctly about the gravity of his situation, and denied that he had killed his employer, but said he remembered striking him. He had thought of killing other employers and persons, just to stop the persecution by telephoning about him. He thought if he should kill some one it would be a lesson to others to stop the annoyance and the hounding. He justified himself by saying, "A man has a right to protect himself."

The more we study cases of paranoia, the more firmly must we be impressed with the dangerous possibilities of the disease. In discussing the best method of dealing with these patients we must remember that the first duty of the state is its own protection and the protection of its different communities, and that the verdict of "not guilty" because of insanity, does not mean liberty. We must remember, too, that although the idea goes forth that the insane man is not responsible for his crime, the erroneous impression is abroad that the lunatic goes "scot free." I cannot speak for every state, but certainly in the State of New York a verdict of "not guilty, because of insanity" in a case of murder is practically the same as a sentence for murder in the second degree.

The public must be educated to the fact, that the man does not get his liberty by the verdict of not guilty when rendered because of insanity; but, on the contrary, the prisoner is taken into an institution where the governor's pardon does not reach him, neither does he ever obtain release when the type of insanity is paranoia, for paranoia is chronic and incurable, tending with the lapse of years to dementia.

In reference to this question, the late Dr. Eskridge, of Colorado wrote: "If the paranoiac who commits a crime in consequence of his delusions is not responsible, what shall be done with him? Every state should have a criminal insane asylum, in which all the insane who have taken human life should be committed for the remainder of their lives. In the absence of a criminal insane asylum they should be imprisoned in a state penitentiary for the rest of their lives. This view in the most recent textbook on legal medicine shows how unsettled is the opinion of the medical profession on this point." I, myself, can only subscribe to this doctrine of perpetual detention as it refers to the paranoiac.

525 Delaware Avenue.

